# P-536. Impact of Maternal RSV Vaccination on Hospitalization Rates in Infants Under Six Months in Buenos Aires, Argentina: A Retrospective Observational Study

**DOI:** 10.1093/ofid/ofaf695.751

**Published:** 2026-01-11

**Authors:** Martin Brizuela, Magali Gonzalez, Tomas Alconada, Sandra Barreiro

**Affiliations:** Hospital General de Agudos Velez Sarsfield, Buenos Aires, Ciudad Autonoma de Buenos Aires, Argentina; Hospital General de Agudos Velez Sarsfield, Buenos Aires, Ciudad Autonoma de Buenos Aires, Argentina; IECS, Ciudad Autónoma de Buenos Aires, Ciudad Autonoma de Buenos Aires, Argentina; Hospital General de Agudos Velez Sarsfield, Buenos Aires, Ciudad Autonoma de Buenos Aires, Argentina

## Abstract

**Background:**

Argentina pioneered RSV immunization in Latin America by implementing maternal RSV vaccination into its National Immunization Schedule on March 1, 2024. This program targets pregnant women between 32 and 36.6 weeks of gestation, aiming to reduce RSV-related hospitalizations and Pediatric Intensive Care Unit (PICU) admissions in infants under six months.

**Methods:**

A retrospective observational study was conducted at a Buenos Aires public hospital, comparing clinical and epidemiological characteristics of infants under six months hospitalized with acute lower respiratory infections (ALRI) between 2022 and 2024. January 1st 2022 to February 29th 2024 was the pre-intervention period, while March 1st to September 30th 2024 was the post-implementation phase. Nasopharyngeal aspirates were tested for respiratory viruses.

**Results:**

Ninety patients were included, 58% male (n=52), median age 4 months. Overall viral infection frequency was 90% (81), with yearly distributions of 97% (29) in 2022, 86% (37) in 2023, and 88% (15) in 2024. RSV was the most prevalent virus (36%,32), with yearly distributions of 37% (11) in 2022, 49% (21) in 2023, and 35% (6) in 2024. Of 17 infants with ALRI in 2024, eight were ineligible for maternal vaccination due to birth outside the recommended gestational window. Seven were born to vaccinated mothers, and two were not vaccinated due to programmatic errors. Among 7 infants of vaccinated mothers, one tested positive for RSV (mother vaccinated two days before premature delivery), five had other viruses, and one had no identified viral infection. On the other hand, among 6 infants with RSV infection, 5 were not vaccinated.

**Conclusion:**

This preliminary analysis suggests a promising trend in RSV prevention through maternal immunization, despite the limited sample size. Timely maternal vaccination appeared protective, with RSV infection observed only in one case with suboptimal timing of vaccination due to premature delivery.

**Disclosures:**

All Authors: No reported disclosures

